# Longitudinal multiple sclerosis lesion segmentation data resource

**DOI:** 10.1016/j.dib.2017.04.004

**Published:** 2017-04-08

**Authors:** Aaron Carass, Snehashis Roy, Amod Jog, Jennifer L. Cuzzocreo, Elizabeth Magrath, Adrian Gherman, Julia Button, James Nguyen, Pierre-Louis Bazin, Peter A. Calabresi, Ciprian M. Crainiceanu, Lotta M. Ellingsen, Daniel S. Reich, Jerry L. Prince, Dzung L. Pham

**Affiliations:** a Department of Electrical and Computer Engineering, The Johns Hopkins University, Baltimore, MD 21218, USA; b Department of Computer Science, The Johns Hopkins University, Baltimore, MD 21218, USA; c CNRM, The Henry M. Jackson Foundation for the Advancement of Military Medicine, Bethesda, MD 20892, USA; d Department of Radiology, The Johns Hopkins School of Medicine, Baltimore, MD 21287, USA; e Department of Biostatistics, The Johns Hopkins University, Baltimore, MD 21205, USA; f Department of Neurophysics, Max Planck Institute, 04103 Leipzig, Germany; g Department of Electrical and Computer Engineering, University of Iceland, 107 Reykjavík, Iceland; h Translational Neuroradiology Unit, National Institute of Neurological Disorders and Stroke, Bethesda, MD 20892, USA

**Keywords:** Magnetic resonance imaging, Multiple sclerosis

## Abstract

The data presented in this article is related to the research article entitled “Longitudinal multiple sclerosis lesion segmentation: Resource and challenge” (Carass et al., 2017) [1]. In conjunction with the 2015 International Symposium on Biomedical Imaging, we organized a longitudinal multiple sclerosis (MS) lesion segmentation challenge providing training and test data to registered participants. The training data consists of five subjects with a mean of 4.4 (±0.55) time-points, and test data of fourteen subjects with a mean of 4.4 (±0.67) time-points. All 82 data sets had the white matter lesions associated with multiple sclerosis delineated by two human expert raters. The training data including multi-modal scans and manually delineated lesion masks is available for download.^1^ In addition, the testing data is also being made available in conjunction with a website for evaluating the automated analysis of the testing data.

**Specifications Table**

**Table:** 

**Subject Area**	Neurology
**More Specific Subject Area**	Neuroimaging
**Type of Data**	Magnetic Resonance Images Specifically: *T*_1_-w MPRAGE
	*T*_2_-w & PD-w DSE
	*T*_2_-w FLAIR
**Data Format**	Raw and Processed
Experimental Factors	None
**Data Source Location**	The Johns Hopkins Hospital,
	Baltimore, MD 21287
**Data Accessibility**	Public download

## **Value of the data**

• This is currently the largest available public database of manually delineated MS lesions.
• All 82 data sets have been manually delineated by two raters.
• A unique multi-modal data set of MS lesion progression covering multiple time-points.
• A public evaluation website allows for the comparison of automated algorithms on the testing data.

## 1 Data

The data presented in this article is related to the research article entitled “Longitudinal multiple sclerosis lesion segmentation: Resource and challenge" [1]. The data consists of magnetic resonance (MR) images (MRI) divided into two cohorts: 1) Training Set; and 2) Test Set. The Training Set consists of five subjects, four of which had four time-points, while the fifth subject had five time-points. The Test Set includes fourteen subjects, ten of which had four time-points, three had five time-points, and one had six time-points. Two consecutive time-points are separated by approximately one year for all subjects. Table 1 includes a demo-graphic breakdown for the training and test data sets. The data does not supply the multiple sclerosis (MS) subtype of the subjects for either the training or the test data. The data is available for download from the Challenge Evaluation Website: http://smart-stats-tools.org/lesion-challenge-2015.

## 2 Methods

**Fig. 1:**
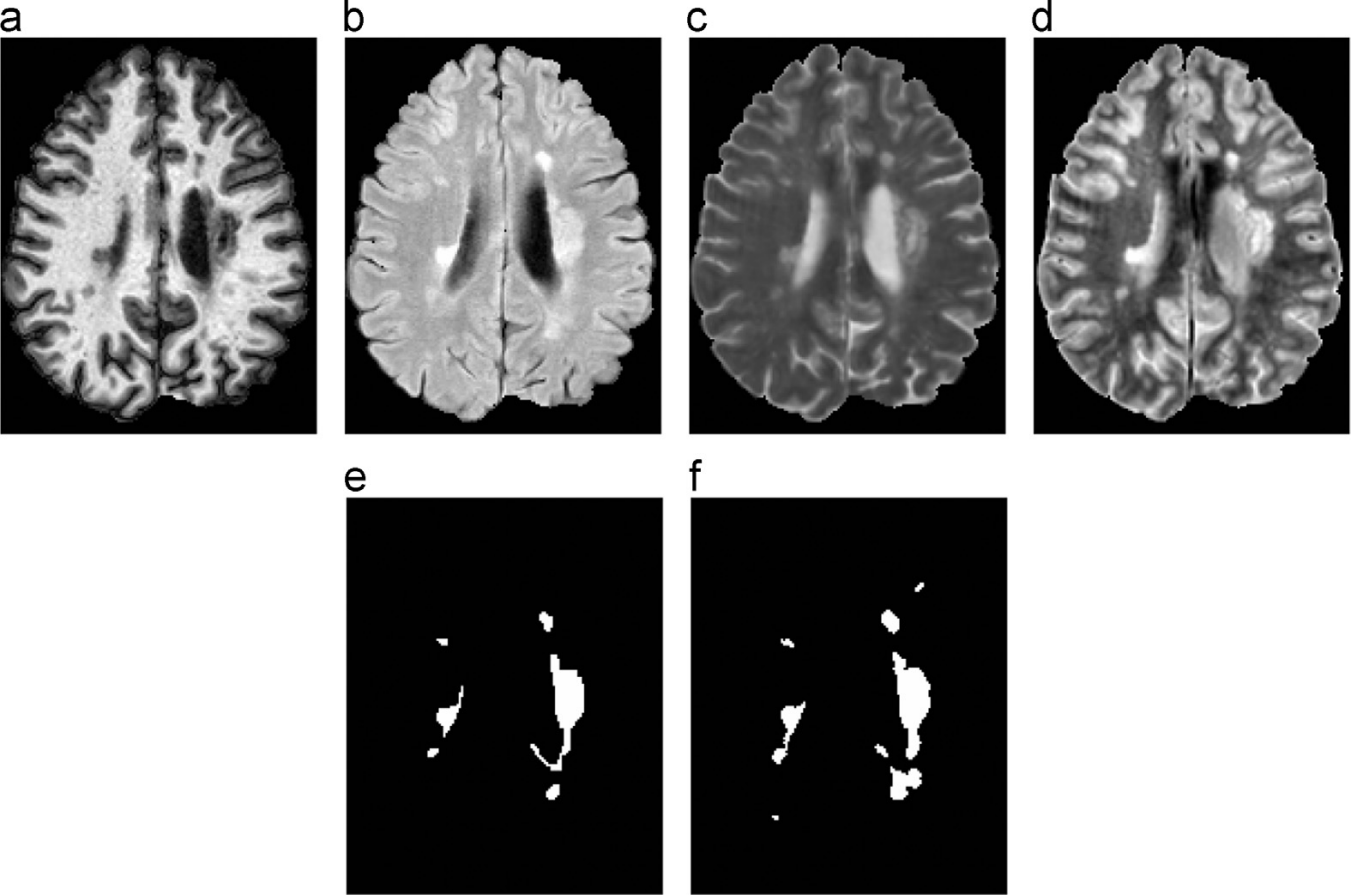
Shown are the preprocessed (a) MPRAGE, (b) FLAIR, (c) *T*_2_-w, and (d) PD-w images for a single time-point from one of the provided Training Set subjects. The corresponding manual delineations by our two raters are shown in (e) for Rater #1 and (f) for Rater #2.

Each scan was imaged and preprocessed in the same manner, with data acquired on a 3.0 T MRI scanner (Philips Medical Systems, Best, The Netherlands) using the following sequences: a *T*_1_-weighted (*T*_1_-w) magnetization prepared rapid gradient echo (MPRAGE) with TR=10.3 ms, TE=6 ms, flip angle=8°, & 0.82×0.82×1.17 mm^3^ voxel size; a double spin echo (DSE) which produces the proton density weighted (PD-w) and *T*_2_-weighted (*T*_2_-w) images with TR=4177 ms, TE_1_=12.31 ms, TE_2_=80 ms, & 0.82×0.82×2.2 mm^3^ voxel size; and a *T*_2_-w fluid attenuated inversion recovery (FLAIR) with TI=835 ms, TE=68 ms, & 0.82×0.82×2.2 mm^3^ voxel size. The imaging protocols were approved by the local institutional review board. Each subject underwent the following preprocessing: the baseline (first time-point) MPRAGE was inhomogeneity-corrected using N4 [2], skull-stripped [3], [4], dura stripped [5], followed by a second N4 inhomogeneity correction, and rigid registration to a 1 mm isotropic MNI template [6]. We have found that running N4 a second time after skull and dura stripping is more effective (relative to a single correction) at reducing any inhomogeneity within the images (see Fig. 1 for an example image set after preprocessing). Once the baseline MPRAGE is in MNI space, it is used as a target for the remaining images. The remaining images include the baseline *T*_2_-w, PD-w, and FLAIR, as well as the scans from each of the follow-up time-points. These images are N4 corrected and then rigidly registered to the 1 mm isotropic baseline MPRAGE in MNI space. Our registration steps are inverse consistent and thus any registration based biases are avoided [7] The skull & dura stripped mask from the baseline MPRAGE is applied to all the subsequent images, which are then N4 corrected again.

For each time-point of every subject׳s scans in the Training Set and Test Set, the following data are provided: the original scan images consisting of *T*_1_-w MPRAGE, *T*_2_-w, PD-w, and FLAIR, as well as the preprocessed images (in MNI space) for each of the scan modalities. The Training Set also included manual delineations by two experts identifying and segmenting the white matter lesions on the MR images.

To facilitate the dissemination of the data and promote the sharing of results we have created a website.^2^ Visitors to the site can see a list of the Top 25 submitted results. Groups interested in running their methods on the data need only register for an account, download the data, and upload their results. The uploader of the results will receive an e-mail within ten minutes detailing the results on a per subject and per time-point basis. The report includes the following computed metrics: Dice, Jaccard, positive predictive value (PPV), true positive rate (TPR), lesion false positive rate (LFPR), lesion true positive rate (LTPR), absolute volume difference (AVD), average symmetric surface distance (ASSD), algorithm and manual lesion volume. The website also provides an overall score; for a given algorithm *A* this is computed as follows,

$$\begin{array}{l} \frac{1}{\left|R\right|}\;\frac{1}{\left|S\right|}\;(\sum_{r\in R}\sum_{\;s\in S}\frac{\mathrm{Dice}\;(M_{r},M_{A})}{8}+\frac{\text{PPV}(M_{r},M_{A})}{8}+\frac{1-\text{LFPR}(M_{r},M_{A})}{4}\\ +\frac{\text{LTPR}(M_{r},M_{A})}{4}+\frac{\text{Corr}(M_{r},M_{A})}{4}), \end{array}$$

where S is the set of all subjects, R is the set of all raters, $M_{r}$ is the lesion mask from rater *r*, $M_{A}$ is the lesion mask for the algorithm *A*, and Corr is the Pearson׳s correlation coefficient of the volumes. This is then linearly normalized by the inter-rater scores between each rater such that the lower inter-rater score has an overall rating of 90. This was designed to mimic the scoring of the 2008 MICCAI MS Lesion challenge [8].

## Figures and Tables

**Table 1:** Demographic details for the training data and both test data sets. The top line is the information of the entire data set, while subsequent lines within a section are specific to the patient diagnoses. The codes are RR for relapsing remitting MS, PP for primary progressive MS, and SP for secondary progressive MS. N (M/F) denotes the number of patients and the male/female ratio, respectively. Time-points is the mean (and standard deviation) of the number of time-points. Age is the mean age (and standard deviation), in years, at baseline. Follow-up is the mean (and standard deviation), in years, of the time between follow-up scans.

**Data set**	**N (M/F)**	**Time-points mean (SD)**	**Age mean (SD)**	**Follow-up mean (SD)**
**Training**	5 (1*/*4)	4.4 (±0.55)	43.5 (±10.3)	1.0 (±0.13)
**RR**	4 (1*/*3)	4.5 (±0.50)	40.0 (±7.6)	1.0 (±0.14)
**PP**	1 (0*/*1)	4*.*0	57*.*9	1*.*0 (±0*.*04)
**Test**	14 (3*/*11)	4.4 (±0.63)	39.3 (±8.9)	1.0 (±0.23)
**RR**	12 (3*/* 9)	4.4 (±0.67)	39.2 (±9.6)	1.0 (±0.25)
**PP**	1 (0*/*1)	4.0	39.0	1.0 (±0.04)
**SP**	1 (0*/*1)	4.0	41.7	1.0 (±0.05)

## Footnotes

2 The data and evaluation website is: http://smart-stats-tools.org/lesion-challenge-2015.
