# Supplementary material for: Longitudinal multiple sclerosis lesion segmentation data resource
Source: Data Brief. 2017 Apr 8;12:346–50. doi: 10.1016/j.dib.2017.04.004 (PMC5412004; doi:10.1016/j.dib.2017.04.004)
Supplement: Supplementary file 1 — Supplementary material [file mmc1.pdf]

February 1, 2017

Hao-Ran Wang PhD
Editor-in-Chief, Data In Brief

Dear Dr. Wang,

Please find enclosed our paper titled *Longitudinal Multiple Sclerosis Lesion Segmentation Data Resource*, for submission to Data In Brief. The authors of this paper declare that they have no known conflicts.

Thank you for your consideration.

Sincerely,

Aaron Carass,
Department of Electrical and Computer Engineering,
The Johns Hopkins University,
3400 N. Charles Street, Baltimore, MD 21218
